# MeDIP Real-Time qPCR has the Potential for Noninvasive Prenatal Screening of Fetal Trisomy 21

**Published:** 2017-02-15

**Authors:** Mohammad Kazemi, Mansoor Salehi, Majid Kheirollahi

**Affiliations:** 1 *Department of Genetics and Molecular Biology, School of Medicine, Isfahan University of Medical Sciences, Isfahan, Iran.*; 2 *Medical Genetic Center of Genome, Isfahan, Iran.*; 3 *Pediatric Inherited Diseases Research Center, Research Institute for Primordial Prevention of Noncommuni-cable Disease, Isfahan University of Medical Sciences, Isfahan, Iran.*

**Keywords:** Down syndrome, trisomy 21, prenatal diagnosis, cell-free fetal DNA; noninvasive prenatal testing; prenatal genetic screening

## Abstract

This study aimed to verify the reliability of the 7 tissue differentially methylated regions used in the methylated DNA immunoprecipitation (MeDIP) real- time quantitative polymerase chain reaction (real-time qPCR) based approach of fetal DNA in maternal blood to diagnosis of fetal trisomy 21. Forty pregnant women with high risk pregnancy who were referred after first or second trimester screening tests, were selected randomly. For each sample whole DNA extraction (mother and fetus), fragmentation of DNA, immunoprecipitation of methylated DNA and real- time qPCR using 7 primer pairs was performed. D-value for each sample was calculated using the following formula D = -4.908+ 0.254 X_EP1_+ 0.409 X_EP4_+ 0.793 X_EP5_+ 0.324 X_EP6_+ 0.505 X_EP7_+ 0.508 X_EP9_+ 0.691 X_EP12_. In all normal cases, D value was negative, while it was positive in all trisomy cases. Therefore, all normal and trisomy 21 cases were classified correctly which correspond to 100% specificity and 100% sensitivity for this method. The MeDIP real-time qPCR method has provided the opportunity for noninvasive prenatal diagnosis of fetal trisomy 21 to be potentially employed into the routine practice of diagnostic laboratories.

Down syndrome (DS) is the most prevalent genetic disease worldwide affecting about one in every 750 live births in all populations and is considered to be the most frequent etiology of intellectual disability (1-4). DS is caused by trisomy of whole or part of chromosome is accompanied with a large amount of health and social costs for patients and their families (5). It is coupled with many health issues, including mental retardation, congenital heart defects, gastrointestinal anomalies, audiovestibular and visual impairment, hematop-oietic disorders, early-onset Alzheimer disease and many other health problems (1, 2, 6, 7). While most fetal aneuplordy leads to miscarriage, trisomy 21 has the maximum survival rate. Due to the highest survival rate, the prenatal detection of fetal trisomy 21 is one of the commonest reasons for referral of women for prenatal diagnosis (8, 9). The incidence of births of children with DS rises with the age of the mother. Screening for DS is an important part of routine prenatal care. Prenatal diagnosis was presented in the 1970s with the major aim of detecting common aneuploidies such as trisomies 21, 18 and 13 (7, 10). Screening tests like the first trimester combination test are nowadays accessible to all pregnant women. Risk calculation is based on maternal age, nuchal translucency (NT) measurement by sonography and two serum markers: free beta hCG (free β-hCG) and pregnancy-associated plasma protein-A (PAPP-A). The test properties are rather good with a detection rate of almost 85– 90% with a false-positive rate of 5–9% (9, 11).

Following a positive prenatal screening test, women are usually recommended to perform fetal karyotyping, which is considered as the gold standard to confirm the presence or absence of aneuploidies. Despite that, the main problem of karyotyping is the long period of time needed to achieve definitive results. Other faster and cheaper methods which have been introduced include interphase FISH and QF-PCR, but their main disadvantage is that they do not provide a full graphic demonstration of all chromosomes (12-15). Prenatal genetic diagnosis of DS and other aneuploidies is done using common cytogenetic tests or DNA analysis which needs fetal DNA to be obtained by invasive methods such as chorionic villus sampling (CVS) during the first trimester and amniocentesis during the second trimester (16-18). Even so, all these methods are invasive and associated with risk of fetal loss (12, 19, 20). Therefore, developing a reliable technique for noninvasive prenatal diagnosis (NIPD) of fetal trisomy 21 is very important (8, 21, 22).

Over the last few years, a great quantity of investigation have been accomplished on the development of noninvasive prenatal testing (NIPT) for fetal aneuploidies (23, 24). The discovery of cell free fetal DNA (cffDNA) in the maternal circulation has opened up a new horizon in the field of prenatal care and screening. Detection of chromosomal aneuploidy is a challenging goal in NIPD research (22, 25-27). Due to the high maternal DNA background and the nature of cffDNA in maternal plasma, determination of chromosomes dosage in the fetal genome is very difficult by common methods. To overcome these issues, background maternal DNA interference can be diminished by using molecular signatures present in maternal plasma but originating completely from fetus. The discovery of fetal-specific DNA methylation signatures in maternal blood offered an excellent opportunity to advent and improve new approaches for noninvasive screening testing. Genes that show differential DNA methylation between placental tissues and maternal blood cells have been used as fetal nucleic acid markers (20, 28-31). DNA methylation is a dynamic process and could change during development. It is believed that more than half of tissue differentially methylated regions (TDMRs) are methylated in embryonic tissues and during the differentiation, they undergo de-methylation process.These TDMRs have been used to enrich and assess fetal DNA ratio by using monoclonal antibody for methylated CpGs using MeDIP approach (16, 18, 32, 33).

The aim of this study was to evaluate and validate the methylated DNA immunoprecipitation of fetal DNA in maternal blood for diagnosis of fetal trisomy 21.

## Materials and methods

**Sample collection and processing**

The samples included 40 pregnant women referred between October 2014 to December 2015 to the Medical Genetic Center of Genome (Iran, Isfahan). All pregnant women agreed to participate in the study and signed an informed consent. The study was approved by the Bioethics Committee of Isfahan University of Medical Sciences. The experimental procedure was followed as previously described with some modifications (16, 33). The participants were women with singleton pregnan-cies, between 13 and 21 weeks of gestation. All participants underwent invasive prenatal diagnosis by CVS or amniocentesis followed by FISH or chromosomal analysis. Briefly, for each pregnant woman 4 ml of peripheral blood was collected on EDTA and then aliquoted into four 1.5 ml tubes and stored at −80°C within 4 h of collection until further use.

**Extraction and fragmentation of DNA**

DNA was extracted from 400 l of peripheral blood sample via QIAamp DNA blood mini kit (QIAGEN) according to the manufacturer's instruction. Subsequently the DNA was quantified using a UV spectrophotometer at 260 and 280 nm and 5 g of the DNA was sheared by sonication at 100% power for 20 min using a WiseClean WUC Digital Ultrasonic (WUC-D06H) into fragment sizes of 100 to 500 bp. Verification of sheared DNA was done by electrophoresis on 1.5% agarose gel.

**MeDIP-real time qPCR**

Sonicated DNA was processed using the MeDIP methodology for immunoprecipitation of hypermethylated fragments (Diagenode’s MagMeDIP kit) according to the manufacturer’s instruction. Finally, real-time qPCR was carried out on an input and immunoprecipitated fragments for the selected 7 differentially methylated regions (DMRs) on chromosome 21 and 2 control regions (hypermethylated region on chromosome 13 and hypomethylated region on chromosome 22) as described previously (33). The real- time PCR was performed with specific primers (Table 1) and Maxima SYBR Green/ ROX qPCR Master Mix (Thermo Fisher Scientific) using StepOne Plus real-time PCR system (Applied Biosystems, USA). Amplification conditions were: first denaturation and enzyme activation at 95 C for 10 min, followed by 40 cycles of amplification at 95 C for 15 s and 60 C for 1 min. The reactions were performed in triplicate. Initially 6 maternal peripheral blood samples with known karyotype (normal pregnancies) were used to calculate the median for normalized □Ct. The ratio value for each of the DMRs was calculated using the median of normalized □Ct obtained from known samples. Finally, D value amount for each unknown sample was calculated using the following formula (33):

ΔCT^PB Normal^= CT^PBNormal Input^- CT^PBNormal IP^

ΔCT^PB T21^=CT^PBT21Input^- CT^PB T21IP^

Where IP correspond to Immunoprecipitated and PB represent the peripheral blood.

Norm ΔCT value ^PBNormal^= E^ΔCTPB Normal^

Norm ΔCT value ^PB T21^= E^ΔCTPB T21^

Where E= 10^[-1/slope]^ and Norm= Normalized

Ratio value ^sample; DMR^= Norm Δ^CTPB Sample(Normal or T21)^/Median (Norm ΔCT^PB Normal^)

D= -4.908+ 0.254 X_EP1_+ 0.409 X_EP4_+ 0.793 X_EP5_+ 0.324 X_EP6_+ 0.505 X_EP7_+ 0.508 X_EP9_+ 0.691 X_EP12_

where X_EPi _is fraction value for each EP marker (33).

**Table 1:** Primers used for real-time PCR (16)

Region	Primer Name	Sequence	Amplicon size (bp)
EP1	***EP1-F***	5’-GCTGGACCAGAAAGTGTTGAG-3’	149
	***EP1-R***	5’-GTGTGCTGCTTTGCAATGTG-3’	
EP4	***EP4-F***	5’-CTGTTGCATGAGAGCAGAGG-3’	95
	***EP4-R***	5’-CGTCCCCCTCGCTACTATCT-3’	
EP5	***EP5-F***	5’-TGCAGGATATTTGGCAAGGT-3’	127
	***EP5-R***	5’-CTGTGCCGGTAGAAATGGTT-3’	
EP6	***EP6-F***	5’-TGAATCAGTTCACCGACAGC-3’	104
	***EP6-R***	5’-GAAACAACCTGGCCATTCTC-3’	
EP7	***EP7-F***	5’-CCGTTATATGGATGCCTTGG-3’	127
	***EP7-R***	5’-AAACTGTTGGGCTGAACTGC-3’	
EP9	***EP9-F***	5’-GACCCAGACGATACCTGGAA-3’	110
	***EP9-R***	5’-GCTGAACAAAACTCGGCTTC-3’	
EP12	***EP12-F***	5’-ATTCTCCACAGGGCAATGAG-3’	128
	***EP12-R***	5’-TTATGTGGCCTTTCCTCCTG-3’	
*HYP113*c	***HYP113*** **c-F**	5’-CAGGAAAGTGAAGGGAGCTG-3’	79
	***HYP113*** **c-R**	5’-CAAAACCCAATGGTCAATCC-3’	
*U122*d	***U122*** **d-F**	5’-AAGGTGCCCAATTCAAGGTA-3’	104
	***U122*** **d-R**	5’-CTTCCCCACCAGTCTTGAAA-3’	

## Results

The ratio values obtained from the 7 selected DMRs (Table 2) were applied to the prediction equation for each sample separately to calculate the D value and determine their status (normal or trisomy 21) (Tables 3 to 5). Cases that gave a D value above zero (cutting point) were classified as “trisomy 21” and below zero was classified as “normal”. A total of 26 cases were classified as normal whereas the remaining 14 cases were classified as trisomy 21 (Table 3). The ratio values and D values obtained for normal and trisomy 21 samples were compared in Table 4. Statistical evaluation of the diagnostic efficiency of the discriminant analysis function, using this method showed a perfect classification for all normal and trisomy 21 cases resulting in a sensitivity and specificity of 100%. Karyotyping of the samples confirmed the above findings (Table 2).

## Discussion

The development of a NIPD technique for fetal trisomy 21 without carrying risk for the pregnancy is a promising research area in prenatal diagnosis (21, 34). The major challenge for the development of NIPD using cffDNA is the limited amount and fragmented structure of cffDNA in the maternal circulation. Over past few years, significant advances have been made for the enrichment and analysis of cffDNA. Nonetheless, most of these techniques are time consuming, laborious or difficult to implement on a large scale (25, 34). Currently, two methods have been developed and validated with almost 100% accuracy. The first method is based on next generation sequencing and the other one is based on MeDIP real-time qPCR. Several reports have shown that application of MeDIP in combination with real-time qPCR using maternal peripheral blood permits prenatal noninvasive detection of trisomy 21. Papageorgiou et al. in 2011 showed that the methylation ratio of normal and trisomy 21 cases for 12 selected DMRs could diagnose 14 trisomy 21 and 26 normal cases indicate 100% specificity and 100% sensitivity of the approach (16). Tsaliki*et *et al. in 2012 confirmed and evaluated this technique for noninvasive prenatal diagnosis of trisomy 21 in larger scale on 175 samples with 99.2% specificity and 100% sensitivity of the approach (33). In another study performed on 10 samples in 2013, Gorduza et al. showed that this approach could detect trisomy 21 cases with 100% specificity (19). The results of our research are in line with those of previous studies and corroborate the high sensitivity and specificity of this method for detection of trisomy 21.The main advantage of this method compared to the next generation sequencing technology is that, this approach could be accessible in all basic diagnostic laboratories as it requires no major and exclusive infrastructure, and is technically easier and less expensive. Moreover, this method will be able to offer results in less than 3 working days (16).

**Table 2:** Ratio values obtained for 40 samples

**Sample**	**Status**	**EP1**	**EP4**	**EP5**	**EP6**	**EP7**	**EP9**	**EP12**
1	TRISOMY	1.398713031	1.926523788	1.33403822	6.712266515	0.054477337	0.749292024	1.12537046
2	TRISOMY	80.81213255	8.975547844	19.84770098	26.97591518	0.701687224	0.037296912	4.588110062
3	NORMAL	3.140730166	0.377879982	1	0.020964613	2.487850856	1.845101267	1
4	NORMAL	0.748775074	2.35169821	0.068878378	0.407612116	0.440901253	0.08029923	0.873795923
5	TRISOMY	3.812401332	1.994462503	1.468354179	1	1.329422798	1	1.12654114
6	NORMAL	0.000217214	0.000752408	0.000812978	0.00026385	0.000352914	0.000547636	0.000574424
7	TRISOMY	0.930836701	4.597979392	0.992060493	11.53703023	0.311412768	1.003610869	0.164709994
8	TRISOMY	2.186464614	1.486582984	1.929865094	21.499035	0.543706507	0.395349362	0.366021424
9	TRISOMY	3.143997347	1.094293701	1.055919608	13.54041452	2.960621374	0.799516659	4.254530692
10	TRISOMY	3.725928527	0.47467106	0.561944673	8.563496658	3.523233276	0.33662168	1.5888688
11	TRISOMY	8.745858457	0.371645746	1.929865094	21.499035	0.135926627	0.395349362	0.366021424
12	TRISOMY	2.156512383	1.829817797	2.526254524	5.095063052	2.545942789	0.730420924	2.047693801
13	TRISOMY	8.773980448	1.145676711	1.480208083	28.44099435	10.39198969	3.655325801	1
14	NORMAL	1.148300315	1	1.069102368	2.484232152	0.595387154	0.619810886	0.546957257
15	NORMAL	0.000108882	0.000159179	9.11287E-05	1.59383E-05	0.000591521	0.037645925	0.03926866
16	NORMAL	1	2.291194604	1.834898169	0.858208443	2.001386775	0.81966142	0.150235744
17	NORMAL	0.329739814	1.918661226	0.224082207	1.151249077	0.00070436	0.008151041	0.162318574
18	NORMAL	2.651116373	0.012242144	1.462564247	2.305692308	0.966539099	0.961394197	1.153485605
19	TRISOMY	1.318228073	3.487032958	1.631840441	28.58526976	3.74223459	6.765516134	3.732131966
20	NORMAL	2.740805592	2.07915887	1.158694309	1.247638519	1.094217853	1	0.668963777
21	TRISOMY	7.318763871	6.480027789	1.054456807	17.16264495	1.841651394	0.78089474	1.519924856
22	NORMAL	4.122730053	2.279946545	1.718917138	0.007584133	1.034475875	1	0.627201102
23	NORMAL	2.04854559	0.496890547	0.201730342	2.553011436	0.559767643	0.697952132	1.435944511
24	NORMAL	3.120116721	1.216722359	1.24444287	2.73435394	0.766788178	0.596750593	0.33844657
25	TRISOMY	2.103220341	0.927873476	0.511746062	13.63459555	1.946523821	0.395349362	0.33844657
26	NORMAL	0.758173533	1.598811661	0.76286517	0.748565259	2.709262157	0.027760936	0.398044049
27	NORMAL	0.466710555	1.112650121	0.195264285	4.767391153	0.011437377	0.015126269	0.489031737
28	NORMAL	0.570619122	0.812252396	1.858965505	1.971371945	0.990274229	0.525659321	0.947370071
29	NORMAL	0.003987	0.399426	2.562408	0.014733	0.249636	0.689776	1.793776
30	NORMAL	0.673896996	1.02313747	0.460253309	1.063780134	1.77509963	1.128338548	0.690158677
31	NORMAL	0.54699517	0.498615626	0.341628443	3.021363992	2.208244625	1.72931418	0.914465089
32	NORMAL	0.010803181	0.173378871	0.128647917	0.600484953	0.525550025	0.010958544	0.968618189
33	NORMAL	0.375425877	0.734584317	0.101145217	0.462395736	0.698290863	0.008345418	0.004100456
34	NORMAL	0.45081268	0.321078952	0.268036244	0.003491832	0.406098049	0.007229943	0.38315499
35	NORMAL	1.306402992	2.177994031	0.464097554	0.665356752	2.946291143	0.198223512	0.542990928
36	NORMAL	0.503687209	0.117358968	2.111839216	5.653718293	0.246883102	0.01382286	0.005727019
37	NORMAL	2.587215093	0.018161158	0.426465222	0.002518534	0.021345859	0.64743174	0.005143621
38	NORMAL	1.734355926	0.054598306	1.243580586	0.0000128382	0.007105244	1.690910149	0.0000690021
39	NORMAL	2.636090682	0.063592481	0.531263683	0.012097058	0.00041262	0.058834247	0.039609766
40	NORMAL	0.022411764	0.054296387	0.05390265	2.53818988	0.000628456	0.38151191	0.026571068
**Mean**		4.003145281	1.449434464	1.445470785	5.988551466	1.319358826	0.796127543	0.91060985
**Median**		1.358470552	1.011568735	1.027228404	2.138532127	0.699989044	0.60828074	0.58707918

**Table 3:** The specification of samples

**Sample**	**Fetal Status**	**Prediction Value** **(D-Value)**	**Gestational Weeks**	**Fetal Gender**	**Confirmed by**
1	Trisomy	0.65367	17	Female	FISH
2	Trisomy	47.31239	18	Male	FISH
3	Normal	-0.27123	16	Female	Amniocentesis
4	Normal	-2.70204	17	Female	Amniocentesis
5	Trisomy	0.322288	17	Male	Amniocentesis
6	Normal	-4.90605	16	Female	Amniocentesis
7	Trisomy	2.51462	18	Male	Amniocentesis
8	Trisomy	5.479775	16	Female	Amniocentesis
9	Trisomy	6.403729	17	Male	FISH
10	Trisomy	2.500866	20	Female	Amniocentesis
11	Trisomy	6.483923	16	Female	Amniocentesis
12	Trisomy	3.113981	18	Male	Amniocentesis
13	Trisomy	55.8825	15	Female	Amniocentesis
14	Normal	-1.56116	14	Male	FISH
15	Normal	-4.86127	18	Female	Amniocentesis
16	Normal	-0.45287	16	Male	Amniocentesis
17	Normal	-3.37215	21	Female	Amniocentesis
18	Normal	-0.5492	19	Female	Amniocentesis
19	Trisomy	15.31432	16	Male	Amniocentesis
20	Normal	-0.51555	16	Female	Amniocentesis
21	Trisomy	8.37518	15	Male	Amniocentesis
22	Normal	-0.09896	16	Female	Amniocentesis
23	Normal	-1.56781	16	Male	Amniocentesis
24	Normal	-0.82083	16	Female	Amniocentesis
25	Trisomy	2.24684	15	Female	Amniocentesis
26	Normal	-1.55669	16	Male	Amniocentesis
27	Normal	-2.28352	14	Female	Amniocentesis
28	Normal	-0.89621	18	Male	Amniocentesis
29	Normal	-0.99089	15	Female	Amniocentesis
30	Normal	-1.6622	15	Male	Amniocentesis
31	Normal	-0.68975	18	Female	Amniocentesis
32	Normal	-3.59748	16	Female	Amniocentesis
33	Normal	-3.92246	18	Male	Amniocentesis
34	Normal	-3.97498	15	Female	Amniocentesis
35	Normal	-1.13799	21	Male	Amniocentesis
36	Normal	-1.08992	15	Female	Amniocentesis
37	Normal	-3.56119	16	Male	Amniocentesis
38	Normal	-2.59636	18	Female	Amniocentesis
39	Normal	-3.72975	13	Female	CVS
40	Normal	-3.8025	17	Male	Amniocentesis
Min		-4.90605	13		
Max		55.88245615	21		
MEAN		3.581176892	16.61904762		
MEDIAN		-1.57612	15.24471		

**Table 4:** The comparison of ratio and D values obtained for normal and trisomy 21 samples

**Normal**					**Trisomy 21**			
	Min	Max	Average	Median	Min	Max	Average	Median
EP1	0.000109	4.12273	1.248103	0.748775	0.000108882	4.122730053	1.248102725	0.748775074
EP4	0.000159	2.351698	0.858713	0.498616	0.000159179	2.35169821	0.858712697	0.498615626
EP5	0.0000911	2.562408	0.796095	0.464098	9.11287E-05	2.562408	0.796095449	0.464097554
EP6	0.0000128	5.653718	1.30727	0.748565	1.28382E-05	5.653718293	1.307270292	0.748565259
EP7	0.000353	2.946291	0.842427	0.559768	0.000352914	2.946291143	0.842426773	0.559767643
EP9	0.000548	1.845101	0.548169	0.525659	0.000547636	1.845101267	0.548168811	0.525659321
EP12	0.000069	1.793776	0.526149	0.489032	6.90021E-05	1.793776	0.526148993	0.489031737
D Value	-4.90605	-0.09896	-2.14400069	-2.370295	0.322288	55.88245615	14.18725183	5.186462

**Fig. 1:**
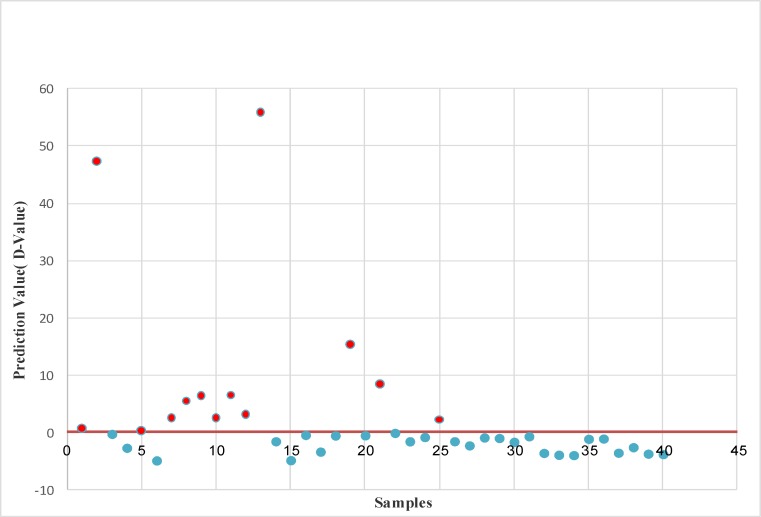
Prediction values (D) derived from the application of the diagnostic formula v1.1 for 40 blind tested cases. The Y axis represents the prediction value D and the X axis shows samples

As different ethnic groups may have different DNA methylation patterns and this could influence MeDIP-qPCR results, the main purpose of the present research was to evaluate and assess an optimized condition for NIPD of fetal trisomy 21 using cffDNA in maternal blood in Iran. We found that our results were in accordance with previous studies and this method is usable for screening in Iran. Furthermore, many different studies will need to be implemented to support the introduction of a new diagnostic strategy into the clinical practice of prenatal diagnostic laboratories.

This approach has provided the opportunity for NIPD of fetal trisomy 21 into many diagnostic laboratories (19, 35). Although the present study is based on a small sample of participants and data from more samples will be of help, our results confirm that this technology could be effective for screening trisomy 21 in pregnant women and could be applied in clinical practice.

## Conflict of Interest

The authors declared no conflict of interest.
